# Risk factors for readmission in schizophrenia patients following involuntary admission

**DOI:** 10.1371/journal.pone.0186768

**Published:** 2017-10-26

**Authors:** Yu-Yuan Hung, Hung-Yu Chan, Yi-Ju Pan

**Affiliations:** 1 Taoyuan Psychiatric Center, Ministry of Health and Welfare, Taoyuan, Taiwan; 2 Department of Psychiatry, National Taiwan University Hospital and College of Medicine, National Taiwan University, Taipei, Taiwan; 3 Department of Psychiatry, Far Eastern Memorial Hospital, New Taipei City, Taiwan; 4 School of Medicine, National Yang-Ming University, Taipei, Taiwan; Maastricht University, NETHERLANDS

## Abstract

**Background:**

Individuals with schizophrenia who are involuntarily admitted may have poorer prognosis, including higher readmission rates, than those voluntarily admitted. However, little is known about the risk factors for readmission in those schizophrenia patients who are involuntarily admitted.

**Aims:**

We aim to explore the risk factors for readmission in this population.

**Method:**

We enrolled 138 schizophrenia patients with involuntary admission from July 2008 to June 2013 and followed those patients for readmission outcomes at 3 months and at 1 year.

**Results:**

The one-year and 3-months readmission rates were 33.3% and 15.2%, respectively. Unmarried status (adjusted odds ratio (aOR) = 6.28, 95% CI: 1.48–26.62), previous history of involuntary admission (aOR = 4.08, 95% CI: 1.19–14.02), longer involuntary admission days (aOR = 1.04, 95% CI: 1.01–1.07) and shorter total admission days (aOR = 1.03, 95% CI: 1.01–1.05) were associated with increased risk for 1-year readmission. Younger age (aOR = 1.10, 95% CI 1.02–1.18) was associated with increased risk for 3-months readmission.

**Conclusions:**

Unmarried status, prior history of involuntary admission, longer involuntary admission days and shorter total admission days were associated with increased risk for 1-year readmission. Healthcare providers may need to focus on patients with these risk factors to reduce subsequent readmissions.

## Introduction

The involuntary admission and treatment of mentally ill patients are central issues in mental health care, relating to long-lasting ethical debates between mentally ill persons’ need to receive treatment and the massive impact upon their rights and freedom. Despite various policies and efforts to reduce the number of psychiatric inpatients, involuntary admission percentages or rates of psychiatric patients have increased across many countries [1]. In 2000, the proportion of involuntary to total psychiatric hospitalizations, termed the involuntary admission quota, ranged from 3.2% to 26.4% across the European Union [1–3]. However, most studies to date on the distribution and associated factors of involuntary admission have been carried out in the Western countries. Although the results may vary in different areas according to their particular legal and sociocultural backgrounds, there are very few studies providing data about involuntary admissions in Asian countries despite their highly variable results [4, 5].

Across the European Union around 30%-50% of involuntary admissions are reported to be attributed to schizophrenia and related psychotic conditions [1, 2, 6]. More recent evidence in Taiwan also shows a very high proportion, 72%, of involuntary admissions being due to diagnoses of psychotic disorders [5]. Indeed, previous studies have indicated that patients diagnosed with psychotic disorders [7–10], especially schizophrenia [11–13], are more likely to be involuntarily admitted. Furthermore, with schizophrenia, readmission is one of the key indicators for future disease prognosis [14], and individuals with an involuntary admission may have higher readmission rates and be more likely to be readmitted compulsorily in the future [15, 16]. Given the massive impact of involuntary admission on the person’s liberty as well as the huge costs inflicted on the person and society related to subsequent readmissions, data regarding readmission and the related risk factors in this population are urgently needed.

According to previous studies on involuntary admission, being single, living alone, young age, short length of admission, drug non-compliance, prior history of involuntary admission, unwillingness to change legal status from involuntary to voluntary, poor psychosocial support and low satisfaction towards the admission experiences, are all risk factors for readmission [17–21]. However, most of the previous studies enrolled patients with various psychiatric diagnoses, making it very difficult to apply the interpretations of study results on a population with any specific diagnosis [15, 21–24]. Among the few studies which focused on patients with schizophrenia, only the outcomes but not the related risk factors were compared between voluntary and involuntary admissions [15, 25]. Therefore, in this study we investigated the risk factors of readmission after discharge from the index involuntary admission in patients with schizophrenia.

## Methods

### Background

The study was approved by the institutional review board of Taoyuan Psychiatric Center (IRB number: B20141231). Beginning in July 2008, the legal process of involuntary admission was modified in Taiwan. Currently, two psychiatrists are required to initiate the compulsory process when people with severe mental illnesses are at risk of self-harm and/or violence and are unwilling to be admitted for treatment. A committee of Ministry of Health and Welfare (MOHW) of Taiwan would subsequently make the judgement regarding whether the involuntary admission is indicated or not. Accordingly, the use of involuntary admission of patients with severe mental illnesses become more restricted.

### Setting

This study was conducted at the Taoyuan Psychiatric Center (TYPC), a major public, psychiatric hospital in Taiwan. TYPC provided 282 acute psychiatric beds and 380 chronic psychiatric beds, which accounted for approximately 50% of the total acute psychiatric beds in Taoyuan City, which had a population of 2.1 million people. The emergency department of TYPC had more than 250 patient visits per month and conducted most of involuntary admissions in Taoyuan City. The study was approved by the local institutional review board (IRB number: B20141231) in Dec. 2014 and we conducted the study from Mar. 2015 to Feb. 2016.

Because this was a retrospective chart review study, our collection of data from their previous medical records would not influence the patients’ clinical treatment. Also, patient identification was not part of the study. Thus the patients who had their data collected would not incur any increased risk, and it would not damage the rights of these patients if they did not provide written informed consent. Therefore, the need for written informed consent was waived by the ethics committee of the study hospital.

### Subjects

The target population was people diagnosed with schizophrenia or schizoaffective disorder who had been admitted compulsorily to TYPC. We used the TYPC electronic medical information system to locate all patients with involuntary admissions. Patients were eligible if their main diagnosis met the International Classification of Diseases, Ninth Revision, Clinical Modification code of 295.xx. Furthermore, we required that the diagnosis of schizophrenia or schizoaffective disorder was approved by the National Health Insurance Bureau of Taiwan with a catastrophic illness card issued from July 2008 to June 2013. We excluded the involuntary admission episodes in which: 1) the diagnosis was converted to other diagnoses rather than schizophrenia or schizoaffective disorder, 2) there was a discharge due to physical illnesses or legal problems, 3) the patient was transferred to a chronic ward, or 4) the involuntary admission was rejected by the Taiwan committee of MOHW.

Involuntary admissions fulfilling the inclusion/exclusion criteria were set as the index admissions. If the individual had more than one involuntary admission during the study period, all of the admissions were individually considered. The medical charts of these patients were reviewed for the following year after the index involuntary hospitalization to collect their demographic data (gender, age, marital status, living condition, comorbid physical illnesses, and employment status) and clinical information (previous history of voluntary or involuntary admissions before the index admission, reason for the index involuntary admission, comorbid alcohol/substance abuse, length of the index involuntary hospitalization, total number of hospitalization days (including involuntary and voluntary hospitalization) of that index admission, whether the patient converted to voluntary admission during the index admission, time to readmission after the index involuntary admission, whether the patient received a long-acting injection (LAI) during the index admission, referral to homecare program after discharge, and number of restraints used during the index involuntary hospitalization.

### Statistical analyses

The primary aim of the current analyses was to identify risk factors for a 1-year readmission. The secondary aim was to identify whether risk factors for a 3-mont readmission would differ from those of the 1-year readmission. Chi-square tests were employed for comparison of categorical variables and independent *t*-test and analysis of variance to compare continuous variables. Unconditional logistic regression was used to explore the possible risk factors of readmission and calculate the odds ratios. We performed the Homer-Lemeshow goodness-of-fit test to assess adequacy of the multivariate models. All tests were two-tailed and p < .05 was considered significant. Data was analyzed with SPSS version 20 (Chicago, IL, USA).

## Results

Initially, we identified 266 involuntary admissions to TYPC from July 2008 to June 2013. Of these, 145 admissions had diagnoses of schizophrenia or schizoaffective disorder. The other admissions had diagnoses other than schizophrenia or schizoaffective disorder (bipolar disorder, n = 43; psychotic disorder NOS, n = 22; organic brain syndrome, n = 13; delusional disorder, n = 11; alcoholic psychosis, n = 11; substance induced psychotic disorder, n = 9; depressive disorder NOS, n = 8; dementia, n = 3; and autistic disorder, n = 1). The age and gender distributions did not differ significantly between those with schizophrenia/schizoaffective disorder and those with other diagnoses.

Of the 145 admissions with diagnoses of schizophrenia or schizoaffective disorder, seven admission episodes were subsequently excluded for the following reasons: three were discharged due to physical illnesses or legal problems; in two cases, diagnoses were converted to either bipolar I disorder or dementia during the index admission; one patient was transferred to a chronic ward; in one case, involuntary admission was not approved by the committee of MOHW.

Therefore, we reviewed the medical records of 138 involuntary admission episodes for the baseline as well as for the following year after their discharge. In the case of loss to follow-up, we made 16 telephone calls to confirm the patients’ latest clinical conditions. Finally, full data for the 130 involuntary admission episodes were obtained. Five admission episodes were missing data following discharge and 3 were lost to follow-up 3 months after discharge from the index hospitalization. The 138 involuntary admissions were contributed by 126 patients with 8 of them having multiple involuntary admissions.

The mean age of the study subjects (*N* = 138) was 38.7 ± 10.9 years, and 61.6% were male. It was their first psychiatric admission for 35 patients (24.6%). Of the overall sample, 112 (81.2%) patients agreed to convert their legal status to voluntary admission during the index hospitalization. Twenty-one (15.2%) patients were readmitted after 3 months and 46 (33.3%) patients were readmitted within the following year after the index involuntary hospitalization (Table 1).

**Table 1:** Demographic data and clinical characteristics.

Description (n = 138)	
**Gender (male), n(%)**	85(61.6%)
**Age, mean (SD)**	38.73(10.88)
**Physical illnesses, n (%)**	22(15.9%)
**Marital status, n (%)**	
Unmarried*	113(81.9%)
Married	25(18.1%)
**Living status, n (%)**	
Alone	16(11.6%)
Live with family	118(85.5%)
Institutionalized	4(2.9%)
**Jobless, n (%)**	123(89.1%)
**1**^**st**^ **psychiatric admission, n (%)**	35(24.6%)
**1**^**st**^ **involuntary admission, n (%)**	117(84.8)
**Reason for the index involuntary admission, n(%)**	
Self-harm	9(6.5%)
Violence	106(76.8%)
Both	23(16.7%)
**Alcohol abuse, n (%)**	21(15.2%)
**Substance abuse, n (%)**	18(13.0%)
**Converted to voluntary admission during the index admission, n (%)**	112(81.2%)
**Readmission within 1 year, n(%)**	
Yes	46(33.3%)
No	84(60.9%)
Unknown	8(5.8%)
**Readmission within 3 months, n (%)**	
Yes	21(15.2%)
No	112(81.2%)
Unknown	5(3.6%)
**Received LAI, n (%)**	29(21.0%)
**Received mood stabilizers, n (%)**	31(22.5%)
**Homecare referral, n (%)**	20(14.5%)
**Duration of involuntary hospitalization of the index admission, days, mean (SD)**	26.52(24.01)
**Duration of total hospitalization days of the index admission, mean (SD)**	59.58(30.10)
**Number of restraint during the index hospitalization, mean (SD)**	2.98(7.95)

*Unmarried status includes single, divorced and widowed

Abbreviations: LAI: long-acting injectable

In the univariate analysis, we compared the demographic/clinical characteristics with regard to whether the patients had a readmission within the 1-year follow-up (*N* = 130). Individuals who were readmitted had a higher proportion of having prior history of involuntary admission before the index hospitalization (26.1% vs. 10.7%, *p* = 0.023). They also had higher proportion of being unmarried (a combination of single, divorced, and widowed) (93.5% vs. 76.2%, p = 0.014). There were no significant differences in terms of conversion of legal status, use of LAI, length of hospitalization and any other variables (Table 2). In the multivariate model, unmarried status (adjusted odds ratio (aOR) = 6.28, 95% CI: 1.48–26.62), history of involuntary admission before the index hospitalization (aOR = 4.08, 95% CI: 1.19–14.02), long involuntary admission days (aOR = 1.04, 95% CI: 1.01–1.07) and short total admission days (aOR = 1.03, 95% CI: 1.01–1.05) were associated with increased risk of 1-year readmission.

**Table 2:** Comparisons between patients with readmission and non-readmission at 1 year.

	readmission (n = 46)	non-readmission (n = 84)	*p*-value*
**Age, mean(SD)**	35.92(8.68)	38.99(10.67)	0.096
**Gender (male), n (%)**	30(65.2%)	51(60.7%)	0.612
**Unmarried status,**^**a**^ **n (%)**	43(93.5%)	64(76.2%)	0.014
**Living alone, n (%)**	3(6.5%)	9(10.7%)	0.430
**Jobless, n (%)**	42(91.3%)	75(89.3%)	0.714
**1**^**st**^ **admission, n (%)**	10(21.7%)	25(29.8%)	0.324
**Prior history of involuntary admission, n (%)**	12(26.1%)	9(10.7%)	0.023
**Alcohol abuse, n (%)**	8(17.4%)	12(14.3%)	0.639
**Substance abuse, n (%)**	6(13.2%)	12(14.3%)	0.845
**Converted to voluntary admission during the index admission, n (%)**	34(73.9%)	72(85.7%)	0.097
**Received LAI, n (%)**	9(19.6%)	18(21.4%)	0.802
**Received mood stabilizers, n (%)**	11(23.9%)	17(20.2%)	0.626
**Homecare referral, n (%)**	4(8.7%)	15(17.9%)	0.157
**Duration of involuntary admission of the index admission, days, mean (SD)**	31.02(30.28)	22.95(19.27)	0.107
**Duration of total admission of the index admission, days, mean (SD)**	54.78(32.19)	60.39(27.50)	0.297
**Number of physical restraint/seclusion during the index admission, mean (SD)**	3.98(10.49)	2.67(6.49)	0.381

*Compared by independent t-test or chi-square test

^a^Unmarried status includes single, divorced and widowed

Abbreviations: LAI: long acting injectable

In terms of the 3-month readmission (*N* = 133), univariate analysis showed that young age had a higher risk of readmission (OR = 1.10, p = 0.005). People who were readmitted within 3 months had a lower proportion of converting to voluntary admission during the index involuntary hospitalization (61.9% vs. 85.7%, p = 0.025). They also had a lower proportion of being referred to homecare program after the index hospitalization (0.0% vs. 17.0%, p = 0.042) (Table 3). However, in the multivariable regression model, young age (aOR = 1.10, 95% CI 1.02–1.18) was the only factor significantly associated with risk of 3-month readmission. Table 4 shows the risk factors for 1-year and 3-month readmission in the conditional logistic regression models, respectively (Table 4). Since the total 138 involuntary admission episodes were actually contributed by 126 patients, we did another sensitivity analysis recruiting only the 1^st^ involuntary admission episodes of those patients, which yielded similar results.

**Table 3:** Comparisons between patients with readmission and non-readmission at 3 months.

	readmission (n = 21)	non-readmission (n = 112)	*p*-value*
**Age, mean (SD)**	32.3(7.36)	39.2(10.43)	0.005
**Gender (male), n (%)**	14(66.7%)	68(60.7%)	0.607
**Unmarried status,**^**a**^ **n (%)**	20(95.2%)	90(80.4%)	0.098
**Living alone, n (%)**	1(4.8%)	12(10.7%)	0.399
**Jobless, n (%)**	19(90.5%)	101(90.2%)	0.966
**1**^**st**^ **admission, n (%)**	7(33.3%)	28(25.0%)	0.426
**Prior history of involuntary admission, n (%)**	6(28.6%)	15(13.4%)	0.102
**Alcohol abuse, n (%)**	2(9.5%)	18(16.1%)	0.739
**Substance abuse, n (%)**	2(9.5%)	17(15.2%)	0.737
**Converted to voluntary admission state during the index admission, n (%)**	13(61.9%)	96(85.7%)	0.025
**Received LAI, n (%)**	4(19.0%)	23(20.5%)	1.000
**Received mood stabilizers, n(%)**	5(23.8%)	24(21.4%)	0.779
**Homecare referral, n(%)**	0(0.0%)	19(17.0%)	0.042
**Duration of involuntary admission of the index admission, days, mean(SD)**	32.00(25.81)	24.57(23.28)	0.189
**Duration of total admission of the index admission, days, mean (SD)**	51.86(34.56)	59.65(27.77)	0.259
**Number of physical restraint/seclusion of the index admission, mean (SD)**	2.29(3.42)	3.23(8.63)	0.622

*Compared by independent t-test or chi-square test

^a^ Unmarried status includes single, divorced and widowed

Abbreviations: LAI: long acting injectable

**Table 4:** Risk factors for readmission (forward conditional logistic regression model).

Variable	Adjusted Odds Ratio (95% CI)
**1-year readmission**	
Unmarried status^a^	4.91 (1.33–18.12)
Prior history of involuntary admission	3.25 (1.19–8.85)
**3-months readmission**	
Not converted to voluntary admission during the index involuntary admission	3.26 (1.12–9.47)
Young age	1.09 (1.02–1.17)

^a^ Unmarried status includes single, divorced and widowed

## Discussion

To the best of our knowledge, this study may be the first research focusing on the longitudinal risk factors of readmission in schizophrenic patients with involuntary admission from an Asian country. In this study, we investigate the related risk factors by analyzing the two patient groups: readmitted and not-readmitted after the index involuntary hospitalization. People with prior history of involuntary admission before the index one are shown to be more likely to be readmitted within the one-year follow-up. Unmarried status (including single, divorced, and widowed) is a risk factor of readmission. In addition, longer involuntary admission days and shorter total admission days are associated with increased readmission rates.

According to previous studies, the readmission rate after discharge from the index involuntary hospitalization is higher than that from voluntary admission [15]. People who had a first involuntary admission were also more likely to be readmitted compulsorily in the future [23, 26]. In our study, schizophrenic patients who had been compulsorily admitted before the index admission showed a higher readmission rate than those who had not been compulsorily admitted before the index admission. This finding is consistent with prior studies since people who had a previous involuntary admission might infer a population with poorer disease awareness and possibly higher severity in psychotic symptoms [25], which in turn might lead to readmission.

We found that unmarried status is a significant risk factor for 1-year readmission in involuntarily admitted patient with schizophrenia. This is consistent with previous studies that being single is a risk factor of readmission in patients with psychiatric illnesses [27, 28]. Despite some evidence indicating that living with others may mean the psychiatric symptoms are detected earlier, thus leading to a higher readmission rate [22], we found no significant difference in readmission rates based on living conditions. This discrepancy might be partly attributed to the fact that a very high proportion of schizophrenia patients (85.5%) in this study lived with their family, which could be more commonly noted in some Asian societies, compared to Western countries.

Previous studies have shown that shorter period of admission might be related to higher readmission rate in patients with schizophrenia [29, 30]. We found similar results that shorter total admission days would increase risk of readmission (aOR = 1.03, 95% CI: 1.01–1.05). It is possible that shorter treatment period might indicate inadequate treatment that leads to poorer outcome. In our study, we also found that longer involuntary admission days would increase risk of readmission (aOR = 1.04, 95% CI: 1.01–1.07). In contrast to total admission days, a longer period of involuntary admission might not indicate adequate treatment. Instead, it might suggest a more severe or refractory disease course and poorer understanding of the disease as well as less effective psycho-social support.

Some people with involuntary admission may change their legal status to voluntary admission after a period of hospitalization. Although these patients may have less severe disease and better prognosis than people who refuse to change [31], Krivoy et al. failed to find such differences in readmission rates according to change of legal status [20]. In our study, people who changed to voluntary admission during the index hospitalization seemed to have lower readmission rates than people who remained under involuntary admission, but it did not reach statistical significance (OR = 0.47, 95% CI: 0.19–1.16, *p* = 0.097). Young age is known to be a risk factor of readmission in psychiatric patients [32]. In our study, we found that it is a risk factor for readmission at the 3-month follow-up. Some related studies have shown that comorbid substance abuse may further increase the risk of readmission [29, 33], but in this study we did not find such results.

Previous research has shown that use of long-acting injectable medication (LAI) may be potentially a protective factor for psychotic symptom relapse and readmission for schizophrenia patients [34, 35]. However, those prior results may not be fully applicable to this specific population of schizophrenia patients with involuntary admission. In this study, we did not find such protective effect, which could be partly attributable to the fact that only a small proportion of patients received LAI (21%). Besides, further research is needed before we can make any conclusions as we did not compare the current results with the proportions of LAI treatments in the involuntary admissions from other studies.

In addition, good community psychosocial support and referral to community care programs have been shown to reduce the readmission rate [36]. In our study, of the total 19 people who were referred to a homecare program after discharge, none were readmitted within 3 months. However, this was not statistically significant in the regression model, perhaps due to the small sample size. The homecare program referral rate was lower than 15% in this study, which might be related to the difficulty of application to a homecare program in Taiwan. First, the patient must have a catastrophic illness card to be qualified as an applicant. Furthermore, the patient must agree with the application. Nevertheless, the involuntarily admitted patients may have poor understanding of their condition, which might mitigate against treatment advice. Therefore, in Taiwan it is more difficult to refer a patient with involuntary admission to a homecare program after discharge.

Previous studies showed that the readmission rate of patients with schizophrenia was about 48% to 67% after 1.5 to 2.5 years follow-up [25]. In our study, the 1-year readmission rate is 33.3%, slightly lower than other studies. The differences may be due to the shorter follow-up period in this study, differing health insurance systems and distinctive socio-cultural environments across different countries or areas.

There are several limitations in our study. While we focus on the specific population of involuntarily admitted patients with schizophrenia, the small sample size is a limitation. In addition, data regarding the reasons why the patients decided to change their legal status as well as the detailed disease course and severity of psychotic symptoms remain unknown. Furthermore, lack of detailed information or comprehensive evaluation of the family/social support and other relevant factors is another limitation because readmission may be affected by many different factors such as coverage of healthcare insurance system, level of insight, and stigma. Future prospective studies can focus on this domain to explore how these socio-clinical factors may influence the disease course and readmission rate.

In conclusion, patients with prior history of involuntary admission, unmarried status, young age, and short total admission days may have higher risk of readmission. To reduce readmission rates, health care providers can focus on patients with these characteristics and provide them with more comprehensive service programs such as programs to enhance disease insight and socio-family support, as well as case management with active follow-up modalities after discharge.

## Supporting information

S1 File Study protocol in English. (DOCX)

S2 File Data extraction form. (DOCX)

S3 File Study protocol in Chinese. (DOC)
