# Supplementary material for: Risk factors for readmission in schizophrenia patients following involuntary admission
Source: PLoS One. 2017 Oct 26;12(10):e0186768. doi: 10.1371/journal.pone.0186768 (PMC5658080; doi:10.1371/journal.pone.0186768)
Supplement: S1 File — (DOCX) [file pone.0186768.s001.docx]

TAOYUAN PSYCHIATRIC CENTER

**Clinical research proposal**

1. Study name:

Risk factors analysis of 1 year readmission of compulsory admitted schizophrenia patients

1. Introduction：

Compulsory admission has played an important role in modern psychiatry and been practiced worldwide for decades though its corresponding legislation and frequency were varied in different countries. People who had been conducted a compulsory admission usually had a higher risk of self-harm or/and violence, and lack of the consent to hospitalization. It suggested that they had more severity in psychiatric problems, poorer disease insight and therefore a poorer prognosis. Evidence shows that people who had been compulsorily admitted had at least equal or greater risk of longer admission duration, higher readmission rate and more possible to be readmitted compulsorily than people who had been admitted voluntarily. Readmission is one of the important clinical indicators of evaluating disease prognosis in people with schizophrenia . Readmission represents a result of complex combinations of multiple clinical factors such as severity of illness, availability of medical resources, family and social support. To reduce further readmission, risk factors related to future readmission needs to be further clarified.

According to previous studies, being single, living alone, young age, short length of admission, drug non-compliance, ever been compulsorily admitted, unwilling to change legal status during admission, poor psychosocial support, low satisfaction of admission experience, etc. are risk factors for readmission. However, most previous studies which investigated the issue of readmission, they usually focused on voluntary admission, not on compulsory admission. Even some studies focused on compulsory admission, they usually enrolled whole psychiatric patients, not specifically in patients with schizophrenia. There are a few studies focused in schizophrenia, but they only investigated the outcome differences between voluntary and involuntary admissions. There is also little information about the readmission rate after discharge from compulsorily admitted schizophrenic patients. Thus, we conducted this study to investigate the aspect of readmission after discharge from compulsory admission and focused in the population of people with schizophrenia.

We assumed that younger age, single, living alone, and history of compulsory admission will increase the risk of readmission. In contrary, receive long-acting injectable, refer to homecare program, convert legal status to voluntary admission will reduce the risk of readmission.

1. Purpose：
   - 1. To obtain the demographic data of compulsorily admitted schizophrenic patients in psychiatric hospital
     2. To collect the risk factors related to readmission
     3. To understand whether the long-acting injectable (LAI) can reduce the readmission rate
     4. To understand whether converting to voluntary admission during compulsory admission can be a good prognostic factor
     5. To understand the relationship between medication choice and readmission rate
2. Methods：
3. Selection criteria and patients numbers：
   1. Inclusion criteria：

compulsory admissions with main diagnosis met the International Classification of Diseases (ICD-9-CM) code of 295.xx from July 2008 to June 2013.

If the people had been compulsorily admitted for more than once in this period, each admission episode was taken into account.

- 1. Exclusion criteria：

1) the diagnosis was converted to other than schizophrenia/schizoaffective disorder, i.e. dementia, during hospitalization, 2) discharged due to physical illness or legal issue, 3) transferred to the chronic ward during the hospitalization, 4) the compulsory admission was rejected by the committee.

- 1. Number of participants：about 150 patients

1. Study designs and procedure：
   - - 1. We continued to collect the information for one year after their discharge. Patients’ chart was reviewed for their demographic data (gender, age, marital status, living condition, comorbid physical illnesses, and employment status) and clinical information (previous history of voluntary or involuntary admissions before the index admission, reason for the index involuntary admission, comorbid alcohol/substance abuse, length of the index involuntary hospitalization, length of total hospitalization days of the index involuntary admission, whether he/she converted to voluntary admission during the index admission, time to readmission after the index involuntary admission, whether he/she received long-acting injectable (LAI) during the index admission, referral to homecare program after discharge, and number of restraints use during the index involuntary hospitalization).
       2. Primary outcome: one-year readmission
2. Statistical analysis：

We used χ2-tests for analysis of categorical variables and *t*-tests or analysis of variance to compare continuous variables. Forward multivariate Cox Proportional Hazard Regression Model was used to investigate the possible risk factors of readmission. Kaplan-Meier survival analysis was used to compare the readmission rate between patients who had or not been compulsorily admitted before. All tests were two-tails and p<.05 was considered significant. Data was analyzed with SPSS version 20.

1. Protection of the patients’ confidentiality
   - - 1. All data collected is from medical charts and already complete before we apply for this study proposal.
       2. All study investigators will not release any data and have signed confidential promise.
