# Supplementary material for: Risk factors for readmission in schizophrenia patients following involuntary admission
Source: PLoS One. 2017 Oct 26;12(10):e0186768. doi: 10.1371/journal.pone.0186768 (PMC5658080; doi:10.1371/journal.pone.0186768)
Supplement: S2 File — (DOCX) [file pone.0186768.s002.docx]

Demographic data and past history

Study ID： Date of data extraction:

| 1. Gender：□1.male □2.female 2. Birthday：□□year □□month 3. Diagnosis of schizophrenia？□0. No □1. Yes 4. Having other psychiatric diagnosis？□0. No □1. Yes   If yes, the ICD-9 code of the diagnosis _______________________   1. Marital status：□1.Single □2.Married □3.Divotced □4.Widow □5.other/unknown 2. Living status：□1.along □2.with family □3.in institution □4. other/unknown 3. Education:□1.<6 yrs □2. 7-9 yrs □3. 9-12 yrs □4. 12-16 yrs □5. >16 yrs 4. Job：□1.jobless □2.retired □3.full-time □4.part-time □5.other 5. Onset of psychotic symptom: □□ y/o 6. First psychiatric visiting: □□ y/o 7. Is this the first time of psychiatric admission：□0. No □1. Yes 8. Number of previous psychiatric admission: □□ 9. Prior history of involuntary admission：□0. No □1. Yes 10. Reason of involuntary admission：□0.self-harm □1. violence □2.both 11. Alcohol abuse history：□0. No □1. Yes 12. Substance abuse history：□0. No □1. Yes 13. Systemic physical illness？□0. No □1. Yes   If yes, the ICD-9 code of the illness:   1. Family history of psychiatric disorder：□0. No □1. Yes | □  □□ □□  □  □  □□□,□□  □  □  □  □  □□  □□  □  □□  □  □  □□  □  □  □□□,□□  □ |
| --- | --- |

Clinical data

Study ID： Date of data extraction:

| 1. Date of the beginning of involuntary admission：□□□year□□month□□day 2. Date of the end of involuntary admission：□□□year□□month□□day 3. Converted to voluntary admission during the hospitalization：□0. No □1.Yes 4. Date of the end of the index admission：□□□year□□month□□day 5. First OPD visiting day after discharge: □□□year□□month□□day 6. Re-admitted within one year：□0. No □1. Yes 7. Date of readmission：□□□year□□month□□day 8. Last OPD visiting day before readmission：□□□year□□month□□day 9. The readmission was involuntary admission：□0. No □1. Yes 10. Last OPD visiting day before loss follow up：□□□year□□month□□day 11. Received long-acting injectables during the index admission：□0. No □1. Yes   If yes, the name of LAI：_____________________   1. Combination use of mood stabilizer：□0. No □1. Yes 2. Homecare referral：□0. No □1. Yes 3. Number of physical restraint/seclusion of the index admission | □□□□□□□  □□□□□□□  □  □□□□□□□  □□□□□□□  □  □□□□□□□  □□□□□□□  □  □□□□□□□  □  □  □  □□ |
| --- | --- |
