# Supplementary material for: Risk factors for readmission in schizophrenia patients following involuntary admission
Source: PLoS One. 2017 Oct 26;12(10):e0186768. doi: 10.1371/journal.pone.0186768 (PMC5658080; doi:10.1371/journal.pone.0186768)
Supplement: S3 File — (DOC) [file pone.0186768.s003.doc]

衛生福利部桃園療養院

**臨床研究計畫書**

1. 計畫名稱：強制住院之思覺失調症病人一年內再住院之危險因子分析
2. 研究機構：衛生福利部桃園療養院
3. 計畫摘要：

利用病歷回顧方式，針對強制住院之思覺失調症病人之基本資料(如：年齡、性別、婚姻狀況、居住狀況…等)以及臨床資料(如：強制住院天數、住院期間是否轉換身份為健保住院、出院時藥物…等)進行統計分析，試圖找出影響強制住院病人出院後一年內再住院之危險因子。

1. 計畫緣由：

思覺失調症病患之強制住院在世界各國儘管實際執行方式和相關法規不盡相同，但都已行之有年(1)(2)，台灣在民國97年7月新制精神衛生法實行後，對於強制住院的規範已更趨完善。根據各國經驗皆顯示強制住院病人的住院天數、再住院率、再受強制住院的比例都較一般自願住院來得高或至少相當(3)(4)。而分析強制住院病人的資料發現，獨居、過去曾強制住院、上次住院滿意度…等，可能為再住院的危險因子(5)。然而在國內，有關強制住院的研究資料極為缺乏，對於強制住院的病人特質、住院治療期間的情形，以及出院後追蹤情形都不甚明瞭。因此，本研究首先將針對強制住院病人的基本特質進行分析，以取得本土化資料。

最常造成治療失敗的原因是藥物的不順從性(6)，而長效針劑的使用能提供較佳的藥物順從性，進而改善病人預後(7)。此外，根據研究，在強制住院一個月後有39%-71%的病人認為自己被強制住院是正確的，而在住院三個月後，認為該次強制住院是正確的比例提高到46%-86%。顯示在經過治療病情改善後，能有較好的病識感(8)(9)。強制住院的狀態並非是固定不變的，強制住院期間，當病人一旦同意住院，即會變更為自願住院。此類病人通常有較佳的病識感，因此也預期可能會有較好的預後(10)。因此本研究也將針對住院期間是否轉變為自願住院、是否使用長效針劑等因素，來分析是否會影響病人的再住院率。

1. 研究目的：
   - 1. 得知於精神科醫院中，強制住院之思覺失調症病人之基本資料(demographic data)
     2. 蒐集易導致個案再入院之危險因子
     3. 了解長效針劑之使用是否有助於減低再住院率
     4. 了解強制住院期間轉換身份為自願住院者，是否有較好之預後
     5. 了解個案強制住院期間之用藥與再住院率之相關性
2. 研究方法：
3. 受試者選擇標準（納入、排除條件）與數目：
   1. 納入條件：

1.本院於2008年7月至2013年8月31日期間，曾於本院接受強制住院並出院之個案

2.該次住院之出院主診斷為思覺失調症(ICD-9：295.XX)

*若收案期間個案有重複一次以上之強制住院，則每次強制住院皆視為獨立事件納入統計

**自願住院期間才轉換身份為強制住院者亦納入統計。

***追蹤期間於他院精神科住院者，等同再入院事件發生

- 1. 排除條件：

1.出院或追蹤期間主診斷不再為思覺失調症者(ICD-9：295.XX)

2.個案於該次住院期間轉入慢性病房者

3.住院期間轉院者

4.追蹤期間直接入住慢性病房或安養機構者

- 1. 受試者數目：估計約150位

1. 研究設計與進行方法：
   1. 進行方式：利用病歷回顧方式，針對個案之年齡、性別、婚姻狀態、居住狀態、教育程度、工作狀態、發病時間(年)、內外科病史、是否有物質濫用史、是否有其他精神科診斷、強制住院之原因(自傷or傷人)、是否第一次住院、過去是否強制住院、過去住院次數、該次住院天數、住院期間是否轉換身份為健保住院、出院時藥物(是否使用長效針劑、是否使用二代藥物、是否併用情緒穩定劑)、出院後可門診追蹤時間(週)、距再入院前最後一次門診時間(週)、出院至再入院時間(週)、再入院時之身份(強制或住院)…等，進行統計分析。
   2. Primary outcome:一年內是否再入院
   3. 是否有對照組：否
   4. 盲化方式：無
   5. 隨機分派：無
2. 研究之評估與統計方法：
3. 使用SPSS20.0統計軟體。
4. 受試者不同risk factors間之比較，針對類別變項利用chi-squared test，針對連續變項則使用independent t-test(若呈skewed distribution，則用Mann-Whitney U test)
5. 用Kaplan-Meier survival analysis比較不同群組間之再住院率(ex:長效針劑vs.口服藥；強制住院vs.轉健保住院)
6. 後續或追蹤計劃：

未來可參考本次研究結果，加入對照組，設計case control study，以比較強制住院與一般健保住院病人之預後。

1. 研究人力及相關設備需求：
2. 研究人力：

| 類別 | 姓名 | 現任職務 | 本計畫內擔任之工作 |
| --- | --- | --- | --- |
| 主持人 | 洪育遠 | 主治醫師 | 主持人 |
| 研究助理 | 待聘 |  | 回顧病歷填寫譯碼表，並協助鍵入電腦 |
|  |  |  |  |
|  |  |  |  |
|  |  |  |  |
|  |  |  |  |

二、相關設備需求：

| 項 目 名 稱 | | 用 途 及 說 明 | 金 額 | 備 註 |
| --- | --- | --- | --- | --- |
| 無 | |  |  |  |
|  | |  |  |  |
|  | |  |  |  |
|  | |  |  |  |
|  | |  |  |  |
|  | |  |  |  |
|  | |  |  |  |
|  | |  |  |  |
|  | |  |  |  |
|  | |  |  |  |
| 共 計 |  | |  |  |

1. 研究對象權益之保障、同意之方式及內容：

1.所有病歷回溯之個案，皆為例行醫療常規治療之案例。

2.所有病歷回溯之個案資料收集，包括例行醫療及追蹤，皆為在計畫提出日前已完成之病歷。

1. 可能傷害之預防、評估與因應：
   1. 計畫主持人及相關研究人員對個案資料負完全之保密責任。
   2. 於資料分析時，受試者的身分皆以代碼表示，且保證於任何時期絕不揭露受試者個人隱私與基本資料。
2. 預期成果及主要效益：
   1. 經由研究調查，瞭解思覺失調症強制住院病人之背景資料及治療預後。
   2. 根據研究結果，瞭解台灣北區某精神科專科醫院思覺失調症強制住院病人之一年內再入院危險因子，並提出建議，作為日後擬定促進醫療品質計畫之參考。
   3. 從中學習臨床研究之設計與執行，累積臨床研究之經驗。
3. 研發成果之歸屬及運用：

研究成果之歸屬與運用歸研究主持人所有。

1. 研究期間與進度：

|  | 第  一  月 | 第  二  月 | 第  三  月 | 第  四  月 | 第  五  月 | 第  六  月 | 第  七  月 | 第  八  月 | 第  九  月 | 第  十  月 | 第  十一  月 | 第  十二月 | 備 註 |
| --- | --- | --- | --- | --- | --- | --- | --- | --- | --- | --- | --- | --- | --- |
| 準備工作 | ● |  |  |  |  |  |  |  |  |  |  |  |  |
| 資料收集 | ● | ● | ● | ● | ● | ● |  |  |  |  |  |  |  |
| 資料分析 |  |  |  |  | ● | ● | ● | ● | ● | ● | ● | ● |  |
| 撰寫報告 |  |  |  |  |  |  |  |  |  |  | ● | ● |  |
|  |  |  |  |  |  |  |  |  |  |  |  |  |  |
| 預定進度 | 10% |  |  |  |  |  | 50% |  |  | 80% |  | 100% |  |

1. 研究經費來源及其需求：
2. 研究經費來源：院內研究經費
3. 研究經費需求：

| 本年度經費需求：本計畫本年度所需各項經費，請依照「衛生福利部經費補助使用範圍及標準表」詳實編列，各經費項目請務必按照該標準表內所訂之名稱與次序填寫，說明欄內應詳細說明估算方法及用途，並依據會計科目分類。 | | |
| --- | --- | --- |
| 項 目 | 金 額 | 說 明 |
| 病歷回顧及資料處理費 | 70000 | 資料譯碼及鍵入費、電腦使用時間費等各項相關費用計約70,000元 |
| 文具、辦公室用品 | 10000 | 紙張、文具、資料夾、印表機碳粉匣（墨  水匣）、感光筒等費用 |
| 雜支 | 10000 |  |
| IRB審查費 | 5000 |  |
| 總計 | 95000 |  |
|  |  |  |
|  |  |  |
|  |  |  |
|  |  |  |
|  |  |  |
|  |  |  |
|  |  |  |

1. 相關文獻：
2. Dressing H, Salize HJ. Compulsory admission of mentally ill patients in European Union Member States. Soc Psychiatry Psychiatr Epidemiol 2004;39(10):797-803.
3. Salize HJ, Dressing H. Epidemiology of involuntary placement of mentally ill people across the European Union. Br J Psychiatry 2004; 184:163-8.
4. Kallert TW, Glockner M, Schutzwohl M. Involuntary vs. voluntary hospital admission. A systematic literature review on outcome diversity. Eur Arch Psychiatry Clin Neurosci 2008;258(4):195-209.
5. Valevski A, Olfson M, Weizman A, Shiloh R. Risk of readmission in compulsorily and voluntarily admitted patients. Soc Psychiatry Psychiatr Epidemiol 2007;42(11):916-22.
6. van der Post LF, Peen J, Dekker JJ. A prediction model for the incidence of civil detention for crisis patients with psychiatric illnesses; the Amsterdam study of acute psychiatry VII. Soc Psychiatry Psychiatr Epidemiol (2014) 49(2):283–90
7. Thieda P, Beard S, Richter A, Kane J. An economic review of compliance with medication in the treatment of schizophrenia. Psychiatr Serv 2003;54:508-16.
8. Haddad  PM;  Taylor  M;  Niaz  OS:  First-generation antipsychotic long-acting injections v oral antipsychotics in schizophrenia: systematic review of randomized controlled trials and observational studies.  Br J Psychiatry Suppl 2009; 195:S20–S28
9. Priebe S, Katsakou C, Glockner M, Dembinskas A, Fiorillo A, Karastergiou A, et al. Patients' views of involuntary hospital admission after 1 and 3 months: prospective study in 11 European countries. Br J Psychiatry 2010;196(3):179-85.
10. Krivoy A, Fischel T, Zahalka H, Shoval G, Weizman A, Valevski A (2012) Outcomes of compulsorily admitted schizophrenia patients who agreed or disagreed to prolong their hospitalization. Compr Psychiatry 53:995–999
11. O'Donoghue B, Lyne J, Hill M, O'Rourke L, Daly S, Larkin C, et al. Perceptions of involuntary admission and risk of subsequent readmission at one-year follow-up: the influence of insight and recovery style. J Ment Health 2011;20(3):249-59.

衛生福利部桃園療養院

**臨床研究主持人暨協同人個人資料**

94.09.28訂定

■研究主持人 □協同人員

|  | 姓名: 洪育遠 性別: 男 出生年月日: 民國63年8月19日 | | | | | | |
| --- | --- | --- | --- | --- | --- | --- | --- |
| 學 歷 | 學校名稱 | | 在校年月日 | | 主修學科 | | 學位 |
|  | 陽明大學 | | 89.9-95.6 | | 醫學系 | | 學士 |
|  |  |  |  |  |  |  |  |
|  |  |  |  |  |  |  |  |
| 經 歷 | 機關名稱 | | 工作單位 | | 職位 | | 在職年月日 |
|  | 桃園療養院 | | 一般精神科 | | 住院醫師 | | 95/7-99/6 |
|  | 金門醫院 | | 精神科 | | 主治醫師、主任 | | 99/7-102/4 |
|  | 桃園療養院 | | 一般精神科 | | 主治醫師 | | 102/5/1至今 |
| 近五年曾參與之專題研究計劃 | 研究名稱 | | 擔任工作 | | 起迄年月 | | 補助機關 |
|  |  |  |  |  |  |  |  |
|  |  |  |  |  |  |  |  |
| 附送近五年著作與研究報告 | 名 稱 | 刊物名稱 | | 卷 期 | 頁 次 | 出版時間 | 出 版 地 點 |
|  |  |  |  |  |  |  |  |
|  |  |  |  |  |  |  |  |
|  |  |  |  |  |  |  |  |
|  |  |  |  |  |  |  |  |

衛生福利部桃園療養院

**臨床研究計畫執行單位同意證明**

**101.10.03訂定**

| 計畫名稱 | 強制住院之思覺失調症病人一年內再住院之危險因子分析 |
| --- | --- |
| 計畫執行地點 | 衛生福利部桃園療養院 |
| 資料收集方法 | 病歷回顧 |
| **計畫主持人申請單位**：  本單位同意此案向衛生福利部桃園療養院倫理委員會提出申請。  此致  倫理委員會  計畫主持人： 洪育遠 單位：一般精神科 職稱：主治醫師  簽名： 日期：  單位主管：詹宏裕 單位：一般精神科  簽名： 日期： | |
| **執行/收案單位**（同申請單位，本欄位免填）：  本單位同意此案於本單位執行/收案。  此致  倫理委員會  本院科主任或授權代表同意簽署(需由機構首長或授權代表同意簽署)  單位： 職稱：  簽名： 日期： | |
